# Elevated Sensitivity to Tactile Stimuli in Stereotypic Horses

**DOI:** 10.3389/fvets.2019.00162

**Published:** 2019-05-31

**Authors:** Sabrina Briefer Freymond, Déborah Bardou, Sandrine Beuret, Iris Bachmann, Klaus Zuberbühler, Elodie F. Briefer

**Affiliations:** ^1^Agroscope, Swiss National Stud Farm, Avenches, Switzerland; ^2^Faculty of Science, Institute of Biology, University of Neuchâtel, Neuchâtel, Switzerland; ^3^School of Psychology and Neuroscience, University of St. Andrews, St. Andrews, Scotland; ^4^Institute of Agricultural Sciences, ETH Zürich, Zurich, Switzerland

**Keywords:** personality, crib-biting horses, stereotypies, coping styles, β endorphin

## Abstract

Although stereotypic behaviors are a common problem in captive animals, why certain individuals are more prone to develop them remains elusive. In horses, individuals show considerable differences in how they perceive and react to external events, suggesting that this may partially account for the emergence of stereotypies in this species. In this study, we focused on crib-biting, the most common stereotypy displayed by horses. We compared how established crib-biters (“CB” = 19) and normal controls (“C” = 18) differed in response to a standard “personality” assessment test battery, i.e., reactivity to humans, tactile sensitivity, social reactivity, locomotor activity, and curiosity vs. fearfulness (both in novel and suddenness situations). Our analyses showed that crib-biters only differed from control horses in their tactile sensitivity, suggesting an elevated sensitivity to tactile stimuli. We suggest that this higher tactile sensitivity could be due to altered dopamine or endogenous opioid physiology, resulting from chronic stress exposition. We discuss these findings in relation to the hypothesis that there may be a genetic predisposition for stereotypic behavior in horses, and in relation to current animal husbandry and management practices.

## Introduction

Stereotypies are defined as repetitive and invariant behaviors, which are thought to be a consequence of suboptimal environmental or housing conditions. Stereotypic behaviors are often described as abnormal and with no obvious goal or function (1), and are sometimes compared to human developmental, neurological, or psychiatric disorders, such as autism, obsessive compulsive disorders or schizophrenia (2). In animals, stereotypies include locomotor (e.g., “pacing”) and oral (e.g., “sham chewing”; “crib-biting”) behavioral abnormalities, which can be debilitating for individuals, especially if they are expressed extensively.

The causal factors and neurobiological mechanisms underlying stereotypic behaviors are only partially understood (2). A recurrent hypothesis is that sustained “stress” or chronic stress, mainly in the form of restricted and suboptimal living conditions, can lead to the development of stereotypic behaviors in animals (2). At the neurobiological level, the idea is that if animals are prevented from executing some behaviors, then this can facilitate the development of alternative behaviors such as stereotypies, via sensitization of the underlying neural systems involved (3). Indeed, exposition to chronic stress is supposed to trigger the release of β endorphin in the brain, stimulating simultaneously dopamine release in the striatum and activating some part of the *basal ganglia* (4). The *basal ganglia* are thought to constitute the location where neural alterations might take place, and hence to play a key role in the development of stereotypies, particularly within the dopaminergic system (5, 6). Yet, it is still largely unclear why only certain individuals develop stereotypic behaviors, while others remain unaffected (7).

One possible explanation for the susceptibility of some individuals but not others to develop stereotypies despite being exposed to similar environments is the existence of individual differences, or personality. Personality and its various sub-traits, such as temperament, refers to between-individual differences in behavior that are relatively stable across various kinds of situations and over the course of time (8–10). This means that differences between individuals are largely maintained, although behavioral attitudes can evolve with age or with the environment (11). Individual differences (i.e., personality) are thought to result from a combination of nature and nurture influences, that is, from an interaction between neural, genetically inherited system (i.e., temperament) and specific environmental influences linked to current and previous experiences during ontogeny (12, 13). Quantifying individual differences (called phenotypes) is usually done via multivariate analyses, which allow behavioral traits to be grouped into larger categories (e.g., “fearfulness” is defined by a range of behavioral reactions to different fear-inducing situations) (14, 15). The more general goal of personality assessments is thus to establish categories that reflect how these animals behave, perceive, and react to the world beyond individual stimuli or specific situations.

One aspect of personality that might affect predispositions to develop stereotypies are individual differences in motivation to perform specific behaviors. Indeed, stereotypies often develop following the prevention of highly motivated behaviors, such as consummatory acts (16). In captivity, the performance of some highly motivated consummatory behaviors may be impossible. This can result in frustration-related stress and, if sustained or repeated, in stereotypies (17). A classic example is carnivores, which are highly motivated to hunt. In captivity, however, individuals are usually prevented from hunting and, according to the stress-by-frustration hypothesis, are thus prone to develop locomotor stereotypies (18). Another classic example is ungulates, which are highly motivated to engage in food processing over long periods of time. Since this is usually not possible in captivity, it frequently results in oral stereotypies (19, 20). Similarly, within each of these systems, individual differences in the propensity to develop certain types of stereotypies could exist. Specifically, in carnivores and rodents, more active individuals could be more prone to develop locomotor stereotypies (21), while in ungulates, more explorative individuals could be more prone to develop food-related stereotypies (22, 23).

Chronic stress, which can occur when animals face aversive situations over prolonged periods of time (24), is another potential precursor of stereotypies. Individual differences in response to chronic stress could thus also affect propensities to develop stereotypies (25). In particular, a distinction has been made between animals responding proactively or reactively when facing an aversive stimulus (26). Proactively coping individuals tend to escape from or remove aversive stimuli (*fight-or-flight*), whereas reactively coping individuals show no obvious reactions in similar situations (*conservation—withdrawal)* (13). In addition, proactive individuals are generally characterized by higher levels of mobility, aggression, exploration, and persistence than reactive individuals (27). These individual differences in coping styles are also frequently related to underlying physiology (26). Proactive individuals tend to have a lower reactivity of the hypothalamo-pituitary-adrenocortical axis (HPA) but a higher reactivity of the sympatho-adreno-medullary (SAM) axis compared to reactive individuals (26). Because proactive animals act to exert control over their environment, they might be more prone to form routines and, by extension, to fall into stereotypies (7). This hypothesis, however, has not been supported by the physiological results of our previous study, which revealed higher HPA-axis reactivity in stereotyped (crib-biters) compared to control horses, which is more characteristic of reactive individuals than proactive ones (28). In sum, the issue of whether stereotypic behavior can be linked to individual differences, and particularly coping styles in response to chronic stress, has not been resolved.

The domesticated horse is an interesting model to study stereotypies, because horses are often confined individually with limited movement for extended periods of time, and with restricted time to forage (19, 29, 30), which makes them prone to develop stereotypies. Other factors, such as sex, age, breed, type of work (dressage), type of diet, and early experiences (e.g., weaning time and start of training) have been associated with the development of stereotypies in this species (31–37). Horses can express different forms of stereotypies, such as weaving, box walking and crib-biting (38, 39). Crib-biting behavior is the most common form of stereotypy in this species (34). It is an oral stereotypy that consists in grasping a fixed object with the incisors, pulling back and drawing air into the esophagus. Crib-biting is related to another common stereotypic behavior, windsucking, which consists of the same behavioral elements, but without grasping an object. The initiation of these behaviors are thought to be associated with diet and time spent foraging (40, 41). Indeed, crib-biting has been shown to increase on low-forage and high-concentrated diet (19, 37, 42, 43). Horses are adapted to eat forage and chew for the majority of the time (29). Because chewing gives the opportunity to moisten food with alkaline saliva essential for digestion, it has been proposed that wood-chewing may precede the development of crib-biting, as a redirected movement in attempts to stimulate saliva production and reduce the acidity of the stomach (40). Despite the fact that no specific genes have been linked to stereotypies in horses, this behavior has been reported more predominantly in certain pedigrees (44–47). Since genetic differences could imply differences in personality, we suggest that individual variation in behavioral responses could predispose horses to develop crib-biting (25).

Even if several studies have aimed at assessing the personality of horses (48–55), investigations of the personality of stereotypic horses are scarce. The evidence so far seems to suggest that crib-biters are less anxious and show no difference in trainability compared to non-crib-biters (56). However, this low level of anxiety in crib-biting horses might be preceded by an initial increase in anxiety, as revealed by an elevation of dopamine, until the stereotypy is fully-established (57). A recent study did also find a relationship between oral abnormal behaviors (crib-biting and lip-twisting) and the “personality traits” intelligence, cooperation, curiousness, equability, and playfulness (58). Overall, a more thorough investigation of crib-biters' personality is required to investigate if a relationship between horse personality and crib-biting might exist.

The aim of this study was to investigate if certain personality traits could be associated with crib-biting behavior. Our current knowledge of crib-biters' personality is limited to the use of questionnaires (56–58). Here, we aimed to obtain more objective measures, by comparing crib-biting and control horses along five “personality” traits following a previously validated model of tests, relevant for equitation practice [reactivity to humans, tactile sensitivity, social reactivity, locomotor activity, and curiosity vs. fearfulness (both in novel and suddenness situations)] (14, 59). These traits have been shown to appear early in life and remain relatively stable across time and situations (60–63). Any possible links between the five traits and crib-biting behavior could, in the long term, help to rapidly identify horses that are more prone to develop stereotypic behavior. According to our previous results on crib-biters' physiology (28), we predicted these horses to show behavioral characteristics of reactive coping individuals, and hence to be generally less anxious (56, 57), or less prone to express their emotions (26), compared to control horses. We therefore also expected them to interact less with unfamiliar humans (i.e., less bold), to show less locomotor and less exploratory behavior (64, 65). Regarding social reactivity, it has been shown in pigs that reactive coping individuals are more social (66). We thus expected, if the same applies to horses, that crib-biters would show more social reactions. Regarding tactile sensitivity, because low responsiveness to external stimuli has been reported in humans and animals after experiencing chronic stress situations [human (67), horses (68)], we expected crib-biters to display a lower tactile sensitivity. Alternatively, because crib-biting behavior has previously been associated with a decrease in nociceptive threshold and therefore potentially enhanced responses to external stimuli (69), the opposite could be predicted, i.e., higher responses in crib-biters to tactile stimulation compared to non-stereotypic horses.

## Materials and Methods

### Subject and Management Conditions

The present study was carried out on 19 crib-biters and 18 control horses (total = 37 horses) of various breeds, sex (mares, geldings, and stallions) and ages (4–24 years old; Table 1), housed in 19 different farms in Switzerland between September 2013 and February 2014. Except for one control, all horses participated beforehand in a study (performed between April and July 2013) aimed at testing the physiological reaction of crib-biters and non-stereotypic horses in a standard ACTH challenge test (28). Twenty-six horses were privately owned, and 11 were obtained from the Swiss National Stud Farm. All the horses had been at their respective farms for at least 1 year. To be eligible for inclusion in the study, crib-biters were required to have demonstrated crib-biting behavior, according to the horse owners, for a minimum of 1 year. The control group was made up of horses that had never been observed crib-biting or performing other stereotypies by their owners [i.e., weaving, box walking, head tossing nodding (38)]. This grouping was verified later on during the first study (28) and during this study (i.e., crib-biters were all observed crib biting, while control horses were not seen displaying any stereotypy). For each crib-biting horse, we tried to find a control horse that was of similar breed, sex and age, and that was housed in the same conditions (i.e., if possible, on the same farm, Table 1). Horses were housed, either individually or in groups, in single boxes or in boxes with paddocks (Table 1). Routine care of the study animals was provided by the farm/horse owners. All these horses were ridden or had been ridden in the past. The study was approved by the Swiss Federal Veterinary Office (approval number VD 2677 bis; Switzerland). The owners of the horses were provided with a detailed written description of the experiment to be conducted and agreed to the research being carried out on their animals.

**Table 1:** Characteristics of the horses used in the experiment.

**Horses**	**Sex**	**Crib-biters or control**	**Age (years)**	**Breed**	**Housing**	**Place**
1	M	CB	6	Warmblood	Box paddock	c
2	M	CB	22	Criollo	Box	g
3	M	CB	16	Franches-Montagnes	Box	y
4	M	CB	9	Hispano-Arabian	Box paddock	b
5	M	CB	5	Quarter horse	Box	s
6	M	CB	9	Paint horse	Box	r
7	M	CB	5	Paint horse	Box paddock	k
8	G	CB	9	Franches-Montagnes	Box	d
9	G	CB	11	Warmblood	Box	g
10	G	CB	23	Franches-Montagnes	Box paddock	n
11	G	CB	11	Franches-Montagnes	Box	bo
12	S	CB	17	Franches-Montagnes	Box	h
13	S	CB	15	Franches-Montagnes	Box	h
14	M	CB	5	Franches-Montagnes	Box paddock	m
15	G	CB	19	Haflinger	Box paddock	se
16	G	CB	18	Warmblood	Box	a
17	G	CB	7	Unknown origin	Box paddock	v
18	G	CB	10	English thoroughbred	Paddock	d
19	S	CB	11	Franches-Montagnes	Box	h
20	M	C	7	Quarter horse	Box paddock	s
21	M	C	20	Franches-Montagnes	Box	y
22	M	C	14	Warmblood	Loose housing	h
23	M	C	18	Camargue	Box paddock	b
24	M	C	14	Warmblood	Loose housing	h
25	M	C	16	Trotter	Box	h
26	M	C	18	Franches-Montagnes	Loose housing	h
27	M	C	10	Warmblood	Box	g
28	G	C	4	Friso-Arabian	box Paddock	n
29	G	C	24	Unknown origin	box Paddock	v
30	G	C	22	English thoroughbred	Paddock	d
31	G	C	7	Quarter horse	Loose housing	k
32	G	C	6	Franches-Montagnes	box Paddock	di
33	G	C	8	Franches-Montagnes	Box	d
34	G	C	15	Warmblood	Loose housing	h
35	G	C	11	Warmblood	Box	h
36	S	C	17	Franches-Montagnes	Box	h
37	S	C	7	Franches-Montagnes	Box	h

*Sex (M, mare; G, gelding, S, stallion), group (CB, crib-biters; C, controls), age, breed, housing (loose housing, paddock, box), and place (each letter refers to a given farm)*.

### Experimental Procedure

The content of this paper is the first part of a study. The other part, aimed at characterizing the learning capabilities of crib-biters, is being prepared for submission (Briefer Freymond et al., submitted).

**Figure 1:**
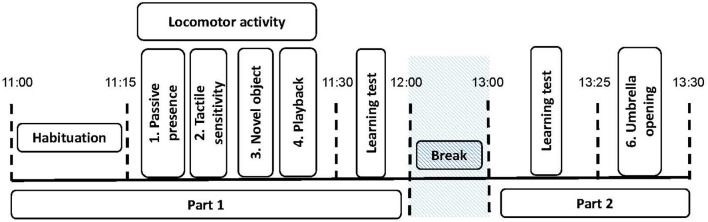
Experimental procedure for the personality tests. The black dotted lines indicate the time at which each period started and ended. The personality tests are indicated (1–5). The different tests are Test 1, *passive presence test (*i.e., *reactivity to human);* Test 2, *tactile sensitivity test (*i.e., *tactile sensitivity);* Test 3, *novel object test (*i.e., *curiosity/ fearfulness)*; Test 4, *playback test (*i.e*., social reactivity)*; Test 5, *umbrella opening test (*i.e., *curiosity/ fearfulness)*. The trait locomotor activity of the horses, was scored as the propensity to demonstrate locomotor activity during the *passive human test*, the *social motivation test*, and the *novel object test*. The learning tests that are indicated are part of another study (Briefer Freymond et al., submitted).

Horses were tested at their home farm in a standardized way. Each horse was subjected to a total of five “personality” tests. The tests were divided in two sessions (Figure 1). Between the two sessions, the horses were returned to their home pen for a break of about 1 h. During this time, in the farms where two horses were tested, the second horse took part in the experiment (Table 1). The procedure, based on preliminary tests performed with 20 pilot horses (different horses as those used in this study), was as follows; at the start of the experiment, the subject was led to and then released by one experimenter (experimenter 1) in a 8 × 10 m delimited arena that was familiar and at the same time, a second experimenter (experimenter 2) started to record the behavior with a camera, Sony Handycam HDR-CX700. After 15 min of habituation to the experimental arena, the first session started and the horse was subjected to four personality tests (about 15 min duration in total, Figure 1). Directly after these tests, another set of learning tests were carried out (Briefer Freymond et al., submitted). A final personality test (about 3 min duration) was performed at the end of the second part (after the learning tests, Figure 1). During the experiment, experimenter 1 recorded the tests with the video and was in charge of preparing the arena for the next tests; whereas experimenter 2 was performing the tests (see details below, in “Personality tests”).

#### “Personality” Tests

**Figure 2:**
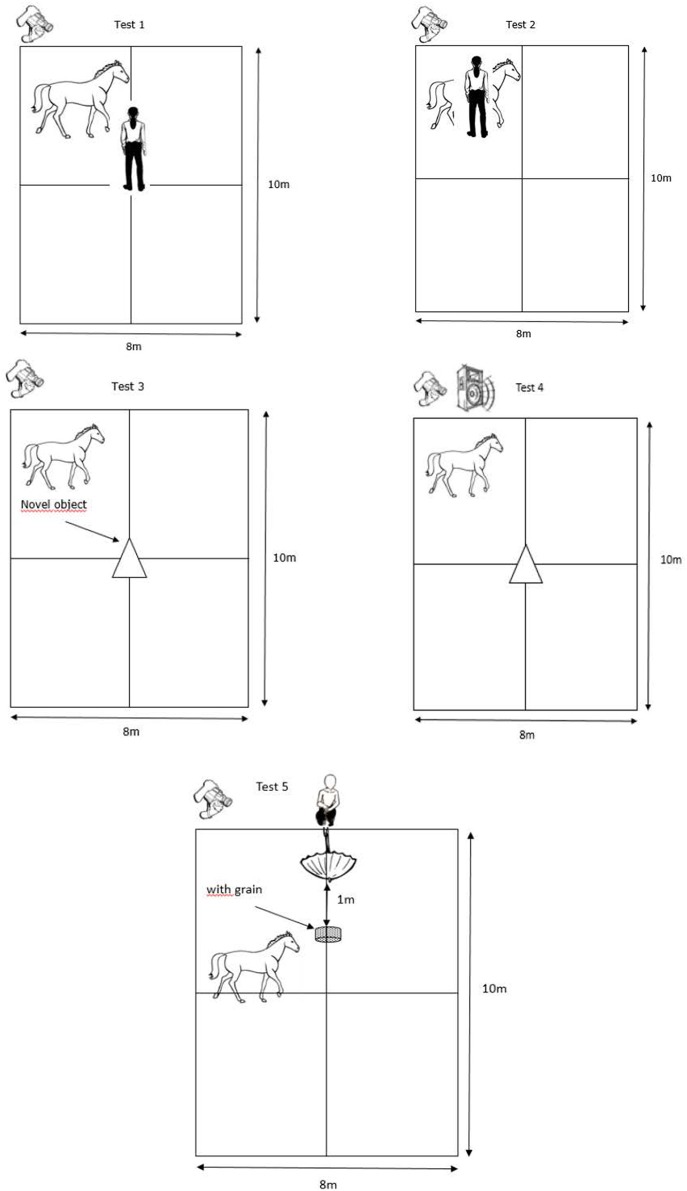
Scheme of the different “personality” tests. The camera is represented at top left. In Test 1 (*passive presence test*), Experimenter 2 is in the middle of the arena. In Test 2 (*tactile sensitivity test*), the Experimenter 2 holds the horse and applies the different filament von Frey. The white triangle in Test 3, (*novel object test)*, designates the unknown object. In Test 4 (*playback test*), the loudspeaker is represented at the top left, next to the camera. In Test 5 (*umbrella opening test*), Experimenter 2, squatted down, holds the umbrella at 1 m from the bucket and 1 m above the ground, the dotted box designates the bucket with food. The black line in the arena designates the arena divided into four sectors to assess locomotor activity (*See Behavioral analyses*).

The horses performed five “personality” tests adapted from Lansade and Bouissou (60) and Lansade et al. (61–63) (Figures 1, 2). The tests were always conducted in the same order. They are presented in the order in which they were conducted. The behavioral measures, based on (60–63), which were scored from a video later on are detailed for each test (see also Table 2). Only those for which the inter-observer reliability (“ICC”) were high and that were expressed by at least 40% of the horses (i.e., 15 horses) are reported. Such cut-off of 40% allowed us to exclude behaviors that were performed by very few animals, and which were hence not representative of the responses of the subjects to our tests (70).

**Table 2:** Definitions of the parameters and the behaviors recorded during the different personality tests (1–5) and to measure the locomotor activity.

**Abbreviation behaviors**	**Definition**	**Tests/personality trait**
“Att” (c)	Proportion of time spent in an attentive state; horse stands still with raised neck and ears pointing toward different stimuli situated outside or inside of the arena (experimenter (Test 1), object (Test 3), camera or outside)	Test 1, 3, 4
“Sr” (c)	Proportion of time spent resting; horse stands still, usually supported by only three legs, with the neck low, the muscles relaxed, the ears to the side, the eyelids closed or half closed, and the lips getting droopy	Test 1
“Sexpl” (c)	Proportion of time spent standing while exploring; horse stands still, with low neck exploring toward different stimuli situated inside of the arena (experimenter (Test 1), object (Test 3), the ground).	Test 1, 3
“Movact” (c)	Proportion of time spent walking active; horse walks with raised neck and ears pointing toward different stimuli situated outside or inside of the arena (experimenter (Test 1), object (Test 3), camera or outside)	Test 1, 3, 4
“Movexpl” (c)	Proportion of time spent walking while exploring; horse walks with low neck exploring toward different stimuli situated inside of the arena (experimenter (Test 1), object (Test 3), the ground)	Test 3
Contact with human, “Conth” or object, “Conto”	Proportion of time spent sniffing, licking or nibbling the experimenter or object, moving the object with the foreleg or the mouth	Test 1, 3
“Whin” (c)	Number of whinnies produced; longest, loudest and most common horse vocalization	Test 4
**Parameters**		
“CAT1” (0–1 m), “CAT2” (>1 m) (s)	Proportion of time spent at (0–1 m) and (>1 m), respectively; estimated distance between the horse and the object or human	Test 1, 3
“React” (b)	Reaction to the tactile filament—trembling or not	Test 2
“Locom” (n)	Sectors entered; number of sectors entered during the Tests 1, 3, and 4	Locom
“Reacum”	Intensity of the reaction to the opened umbrella; nothing (“A”), raises head (“B”), steps back (“C”), jumps back and looks at the umbrella (“D”), jumps back and looks outside (“E”), jumps back and canter (“F”)	Test 5
“Flight”	Flight distance from the opened umbrella; how far the horse moves away from the food bucket (“A” = 0 m; “B” = 0–1 m, “C” = 1–2 m, “D” ≥ 2 m)	Test 5
“Time”	Time until eating in seconds; time to resume eating in the bucket after the umbrella was opened	Test 5
**Control factors**		
“Force”	Five different filament forces (“F”) applied to the skin (“F1” = 300 g, “F2” = 0.6 g, “F3” = 0.02 g, “F4” = 0.008 g, “F5” = 3 g)	Test 2
“Sound”	Sound treatments played to the horses; whinny from a mare, whinny from a gelding, and a control sound (skylark song)	Test 4
“Order”	Order in which the sound treatments were played to the horses	Test 4

*C, continuously recorded or as duration; s, instantaneous time sampling; n, number; b, binary*.

*The crosses indicate which parameters or response variables were recorded for each personality trait. The different tests (i.e., personality trait) are Test 1, passive presence test (i.e., reactivity to human); Test 2, tactile sensitivity test (i.e., tactile sensitivity); Test 3, novel object test (i.e., curiosity/fearfulness); Test 4, playback test (i.e., social reactivity); Test 5, umbrella opening test (i.e., curiosity/fearfulness); Locomotor activity (“Locom”)*.

#### Test 1: Passive Presence Test

This test assesses the propensity of a horse to react to a passive human, i.e., “reactivity to humans.” An unknown experimenter (always the same person; experimenter 2) entered the test pen and settled motionless in the middle of the arena. The horse had the possibility to interact with the motionless person for 3 min (Figure 2). We scored the following behaviors related to the trait “reactivity to humans”; the time the horses spent interacting with the unknown experimenter (“Conth”), the time spent standing attentive (“Att”), the time spent standing resting (“SR”), the time spent standing while exploring (“Sexpl”), the time spent walking active (“Movact”) and the time spent close (0–1 m, “CAT1”) or far (>1 m, “CAT2”) from the unknown person (Table 2).

#### Test 2: Tactile Sensitivity Test

This test assesses the propensity of a horse to react to a greater or lesser extent to tactile stimuli, i.e., “tactile sensitivity.” Experimenter 2 held the horse and applied a “filament von Frey” on its skin (Figure 2). These filaments consist of a hard plastic body connected to a nylon thread, and are calibrated to exert a specific magnitude of force on the skin, ranging from 0.008 to 300 g. Such filaments are commonly used to measure mechanical sensory thresholds in people (through verbal responses) and animals (through behavioral responses) (71). Five different forces were applied, always in the same random arrangement (300, 0.6, 0.02, 0.008, 1 g) perpendicularly to the animal's skin at wither's height, until the nylon thread started to bend (i.e., for the exact location of the application of the filaments, see (68). The interval between the application of each filament was about 30 s. Experimenter 1 recorded directly in binary form the following behavior related to the trait “tactile sensitivity”; trembling of platisma muscle [behavior used by horses to drive away flies, (72); trembling or not, “React”; Table 2].

#### Test 3: Novel Object Test

This test assesses the propensity of a horse to react with fear or curiosity when exposed to a novel situation, i.e., “curiosity/fearfulness.” A novel object (i.e., transparent hose fixed with colorful string) was placed in the middle of the arena, in front of the horse held by experimenter 2. The horse was then released for a duration of 3 min, during which it had the possibility to explore the object (Figure 2). We analyzed the following behaviors related to the trait “curiosity/fearfulness”; the time the horse spent interacting with the novel object (“Conto”), the time spent standing attentive (“Att”), the time spent standing while exploring the ground (“Sexpl”), the time spent walking active (“Movact”), the time spent walking while exploring (“Movexpl”), and the time spent close (0–1 m, “CAT1”) or far (>1 m, “CAT2”) from the novel object (Table 2).

#### Test 4: Playback Test

This test assesses the propensity of a horse to react to a conspecific, i.e., “social reactivity.” This test was adapted from a study including a playback procedure (73). It consisted in measuring the reactions of the subjects to the vocalizations of conspecifics. We used a loudspeaker located next to the camera, on one side of the arena, and played one 2-s whinny from an unknown mare, one 3-s whinny from an unknown gelding, and a control sound (15 s of skylark song, *Alauda arvensis*) (Figure 2). All the sounds were played at similar amplitude, estimated to be normal for the horses (85.2 ± 2.4 dB measured at 1 m using a sound level meter, C weighting; SoundTest-Master, Laserliner, UK). All the horses received the same three sounds, played in a random order, with 10-s silence interval. We analyzed the following behaviors related to the trait “social reactivity”; the vocal response of the horses to each sound (“Whin”), the time spent walking active (“Movact”) and the time spent standing attentive (“Att”) (Table 2).

#### Test 5: Umbrella Opening Test

This test was carried out after a battery of learning tests (Briefer Freymond et al., submitted). It assesses the propensity of a horse to react with fear or curiosity to a sudden situation (umbrella opening), i.e., “curiosity/fearfulness.” This test, in a similar way to Test 3 (*novel object test*), measures fear reactions but this time in situations involving suddenness. A bucket of pellets was placed next to the entrance, with a closed umbrella held at 1 m from the bucket and 1 m above the ground by experimenter 2, who was visible to the horse. Experimenter 1 released the horse and it was free to go eat from the bucket. When the animal was eating with its head in the bucket for more than 3 s, experimenter 2 suddenly opened the umbrella and the chronometer started (Figure 2). The test stopped when the horse resumed eating. The time it took for the horses to come back to eat in the bucket after the umbrella opened was directly recorded (“Time”). A maximum of 300 s was allocated. In case the horse did not come back to eat, the time was fixed at 300 s (Time = 300 s). We scored the following behaviors related to the trait “fearfulness”; the intensity of the reaction using a scale (“React”) (Table 2) and the estimated flight distance of the horses after opening the umbrella (“Flight”) (Table 2). Because of a technical problem with the cameras, we were not able to score the behaviors React and Flight of two control horses in this test.

#### Behavioral Analyses

All tests were video recorded by experimenter 1, who was located outside of the arena (Figure 2), using a Sony Handycam HDR-CX700. From the video of the tests, two different observers (experimenter 2 and another observer, who was blind to the group of the horses since she had not participated in the experiment and scored the videos after renaming them with a code) scored for each test (i.e., all the videos) the behaviors either as occurrence using an instantaneous time sampling method every 10 s (for “CAT1” and CAT2 ; “Point Events”), or continuously as duration (for other behaviors; “State Events”) using the Observer software XT v.11 (Noldus). We then calculated the frequency of occurrence for the Point Events, and the proportion of the total time spent performing the behavior for State Events (Table 2). The last personality trait assessed in the study, locomotor activity of the horses, was scored as the propensity to demonstrate locomotor activity during the *passive human test*, the *social motivation test*, and the *novel object test*. In order to record this personality trait, the arena was divided into four sectors of equal size using tracks made in the sand beforehand (Figure 2). To assess the locomotor activity, experimenter 1 recorded directly the number of times the horses changed sectors (“Locom”) (Table 2). Because of a problem, we were not able to score the activity level of three control horses in this test.

### Statistical Analysis

#### Inter-observer Reliability (ICC)

Inter-observer reliability between the two observers scoring the videos continuously was assessed by intraclass correlation coefficients (ICC). ICC were calculated using a two-way mixed design to assess the absolute agreement between the scores of the two observers (74, 75). ICCs range from 0 to 1, with 0 indicating no agreement and 1 indicating full agreement. Generally, ICCs ≤ 0.40 are considered as poor, those between 0.40 and 0.59 as fair, those between 0.60 and 0.74 as good, and those between 0.75 and 1.00 as excellent (76). We kept for the analysis only the behaviors for which ICCs revealed fair to excellent agreements between the scores of the two observers. To this aim, the time spent at 1–2 m and the time spent at more than 2 m (Table 2), which obtained low ICC (ICC: range = 0.35–0.55) were grouped into one category (CAT2; ICC: range = 0.90–0.93).

#### Behavioral Measures

The statistical analyses were carried out on the behavioral parameters for which the inter-observer reliability (ICC) between the two observers was fair to excellent (ICC: mean ± SD = 0.84 ± 0.14; range = 0.55–0.98). The behavior of the crib-biters (CB) was compared to the behavior of the control horses (C) for each test separately (Tables 2, 3), using linear mixed-effects models (LMM; lmer function, lme4 library), generalized linear mixed models [GLMM; glmer function; lme4 library; multcomp library; (77)], or cumulative link mixed models [CLMM, clmm function in R 3.0.2 (78)]. The different models included as a response variable the behavioral parameters scored (Tables 2, 3). The fixed and control factors are described in Tables 2, 3. To control for repeated measurements of the same subjects, the identity of the horses nested within the farms where they were housed (“Farms”) was included as a random factor for Tests 2 and 4. For Test 1, Test 5, and for the locomotor activity, only Farms was included as a random factor, as there was only one behavioral value for each horse. When significant interaction effects between fixed and/or control factors were found, further *post-hoc* analysis were carried out using further LMMs and GLMMs. Bonferroni corrections were applied to these *post-hoc* tests accordingly.

**Table 3:** Response variables, as well as fixed and control factors used in the different model (LMM and GLMM).

**Response variables**	**Test 1**	**Test 2**	**Test 3**	**Test 4**	**Test 5**	**Locom**
Att	x		x	x		
Sr	x					
Sexpl	x		x			
Movact	x		x	x		
Movexpl			x			
Conth	x					
Conto			x			
Whin				x		
CAT1	x		x			
CAT2	x		x			
React		x				
Locom						x
Reacum					x	
Flight					x	
Time					x	
**Control and fixed factors**						
Group	x	x	x	x	x	x
Force ^*^ Group		x				
Sound ^*^ Group				x		
Order ^*^ Group				x		
Sex	x	x	x	x	x	x
Age	x	x	x	x	x	x
Arena	x	x	x	x	x	x
Farm	x	x	x	x	x	x
Force		x				
Order				x		
Sound				x		

The abbreviations are described in Table 2. The crosses (“x”) indicate which response variables or factors were recorded in the different personality tests (1–5) and to assess the locomotor activity “Locom,” The different tests are Test 1, passive presence test; Test 2, tactile sensitivity test; Test 3, novel object test; Test 4, playback test; Test 5, umbrella opening test. The fixed parameters are the “Group” (“CB” = crib-biters and “C” = controls), the interaction term “

* *” between the filament forces “Force” and Group CB-C (Test 2), the interaction term between sound treatment “Sound” and Group CB-C and between the order of the sound “Order” and Group CB-C (Test 4). The other parameters are control parameters: the sex, age, and farm where the horses were housed (Table 1), whether the arena where the horses were tested was situated outside or indoor “Arena”, Force, Order, and Sound (Table 2)*.

The residuals were checked graphically for normal distribution and homoscedasticity. To satisfy the model assumptions, we used log transformation for “Att” and “Movact” in the *presence passive test*, “Movact” and “Sexpl” in the *unknown object test*, and for “Time” in the *umbrella test (*Table 2). All the parameters satisfying model assumptions were then input into LMMs (lmer function). Some parameters did not meet the statistical assumptions despite transformation. They were thus transformed to binomial data as follows; behavior occurred = 1 or did not occur = 0 for “CAT1” in *novel object test*, “Movact”, “Sexpl,” and “Sr” in *presence passive test*, and “Movact” in *playback test* (Table 2); and value equal or higher than median = 1 or value lower than median = 0, for “Att,” “Conth,” “CAT2” in *novel object test*, “Att” in *playback test* and “Locom” for *locomotor activity* (Table 2). The parameters scored as binomial (“React” and “Whin”), as well as parameters transformed to binomial data, were input into GLMMs fit with binomial family distribution and logit link function (glmer function). In the *umbrella opening test*, in order to compare the intensity of the reaction after opening the umbrella (“Reacum”) and the distance of flight from the open umbrella (“Flight”) (Table 2), we used CLMM (clmm function) (79). To this aim, Reacum was transformed in six distinct categories and Flight in four categories (Table 2).

For the LMMs and GLMMs, a standard model simplification procedure was used to remove each non-significant term until the deletion caused a reduction in goodness of fit (in this case, the term was left in the model). *P*-values were calculated based on Satterthwaite's approximations (anova function, lmerTest package in R). The significance level was set at α = 0.05. Only the results of the fixed factors are described in details in the results.

## Results

### Passive Presence Test

There were no differences between groups CB and C in their time spent interacting with the person (“Conth”), standing attentive (“Att”), standing while resting (“Sr”) and standing while exploring the ground (“Sexpl”) (LMM: effect of Group CB-C on Conth, $X_{1}^{2}$ = 1.71, *p* = 0.19; effect of Group CB-C on Att, $X_{1}^{2}=$ 1.66, *p* = 0.20; GLMM: effect of Group CB-C on Sr, $X_{1}^{2}=$ 0.26, p = 0.87; effect of Group CB-C on Sexpl, $X_{1}^{2}=$ 0.27, *p* = 0.60). There were also no group differences neither in the time spent walking active (“Movact”), nor in the time spent close (“CAT1”) or far (“CAT2”) from the unknown person (LMM: effect of Group CB-C on Movact, $X_{1}^{2}=$ 0.11, *p* = 0.74; LMM: effect of Group CB-C on CAT1, $X_{1}^{2}$ = 0.89, *p* = 0.35; LMM: effect of Group CB-C on CAT2, $X_{1}^{2}\;=$ 0.89, *p* = 0.35).

### Tactile Sensitivity Test

**Figure 3:**
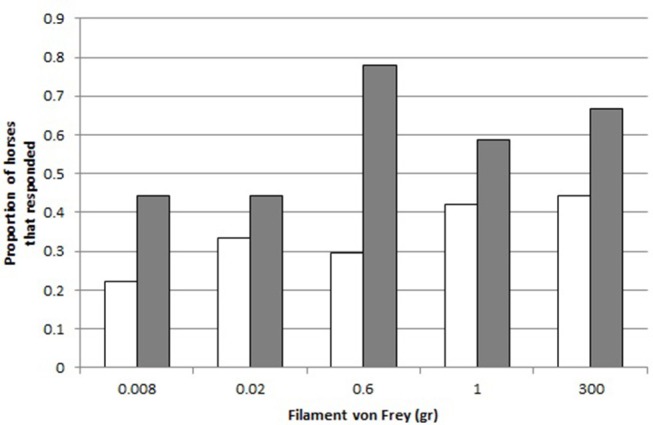
Responses to the different filaments von Frey. Proportion of controls (C, white, *N* = 18) and crib-biters (CB, gray, *N* = 19), respectively, that responded to each Filament von Frey (300, 0.6, 0.02, 0.008, 1 gr.).

A greater proportion of crib-biters reacted to the filament von Frey (“React”) than the control horses (GLMM: effect of Group CB-C on React, $X_{1}^{2}\;=$ 8.14, *p* = 0.004, Figure 3).

### Novel Object Test

There were no differences between groups CB and C in their time spent interacting with the unknown object (“Conto”), standing attentive (“Att”), and standing while exploring the ground (“Sexpl”) (GLMM: effect of Group CB-C on Conto, $X_{1}^{2}$ = 0.30, *p* = 0.58; effect of Group CB-C on Att, $X_{1}^{2}$ = 0.67, *p* = 0.41; LMM: effect of Group CB-C on Sexpl, $X_{1}^{2}$ = 0.67, *p* = 0.41). There were also no group differences, neither in their time spent walking active (“Movact”), in walking while exploring (“Movexpl”), nor very close (“CAT1”) or very far (“CAT2”) from the object (LMM: effect of Group CB-C on Movact, $X_{1}^{2}$ = 0.49, *p* = 0.48; GLMM: effect of Group CB-C on Movexpl, $X_{1}^{2}$ = 0.41, *p* = 0.52; effect of Group CB-C on CAT 1, $X_{1}^{2}$ = 0.005, *p* = 0.94; effect of Group CB-C on CAT 2, $X_{1}^{2}$ = 0.02, *p* = 0.89).

### Playback Test

There were no differences between groups CB and C in their vocal responses to the playbacks (“Whin”) and in the time they spent standing attentive (“Att”) (GLMM: effect of the Group CB-C on Whin, $X_{1}^{2}$ = 2.77, *p* = 0.10; effect of the Group CB-C on Att, $X_{1}^{2}$ = 0.016, *p* = 0.73). There was also no group effect on the time spent walking active (“Movact”) during this test (GLMM: effect of the Group CB-C on Movact, $X_{1}^{2}$ = 2.06, *p* = 0.15).

### Locomotor Activity

There were no differences between groups CB and C in the number of sectors they entered, which reflects locomotor activity (“Locom”) (GLMM: effect of the group CB-C on Locom, $X_{1}^{2}\;$ = 1.74, *p* = 0.18).

### Umbrella Test

There were no differences between groups CB and C in their time taken to resume eating in the bucket after the umbrella was suddenly opened (“Time”) (LMM: effect of the Group CB-C on Time, $X_{1}^{2}\;$ = 0.69, *p* = 0.41). There were also no differences between groups in the intensity of their reaction after the umbrella was opened (“Reacum”) (CLMM: effect of the Group CB-C on Reacum, $X_{1}^{2}\;=$ 0.366, *p* = 0.55). Finally, there was no group effect on the flight distance (“Flight”) (CLMM: effect of the Group CB-C on Flight, $X_{1}^{2}$ = 1.065, *p* = 0.30).

### Control Factors

The type of arena (indoor or outdoor, “Arena”) had a significant effect on Reacum in the *umbrella test*, (CLMM: effect of Arena on Reacum, $X_{1}^{2}\;$ = 4.2, *p* = 0.04), and on React in the *tactile sensitivity test* (GLMM: effect of Arena on React, $X_{1}^{2}$ = 5.36, *p* = 0.02). The age of the horses (“Age”) had a significant effect in the *passive presence test* on CAT1 and CAT2 (GLMM: effect of Age on the CAT1, $X_{1}^{2}\;$ = 6.09, *p* = 0.01; effect of Age on CAT2, $X_{1}^{2}\;=$ 6.09, *p* = 0.01) and on Att (LMM: effect of Age on Att, $X_{1}^{2}\;$ = 9.15, *p* = 0.002). In the *Novel object* test, Age had an effect on CAT2 (GLMM: effect of Age on CAT2, $X_{1}^{2}\;$ = 9.90, *p* = 0.002). The sex of the horses (“Sex”) had a significant effect in the *playback test* on Movact and on Att (GLMM: effect of Sex on Movact, $X_{2}^{2}\;$ = 6.20, *p* = 0.05; GLMM: effect of Sex on Att, $X_{2}^{2}\;$ = 5.99, *p* = 0.05). The strength of the filaments (“Force”) had an effect in the *tactile sensitivity test* on React (GLMM: effect of Force on React, $X_{4}^{2}\;=$ 13.78, *p* = 0.008). The order in which the sound treatments were played to the horses *in the playback test* (“Order”) had an effect on Movact (GLMM: effect of Order on Movact, $X_{1}^{2}\;$ = 6.76, *p* = 0.009). The control factors not mentioned above were not significant and were thus removed from the models during model selection.

## Discussion

Environmental causes responsible for the development of stereotypies are partially known (80), but little is known about why some individuals develop stereotypies and others do not, despite being exposed to the same environmental conditions. Here, we tested the hypothesis that predispositions to stereotypies might be linked to individual differences in behavior (“personalities”), which are in part genetically determined. To this aim, we compared how stereotypic and non-stereotypic horses (controls), responded to a standardized test battery commonly used to assess individual differences in horses (60–63). Based on previous findings (26, 28, 56, 64, 65, 68, 69), we expected crib-biters to show behavioral characteristics of reactive coping individuals, namely to be less anxious (e.g., fearful), to interact less with unfamiliar humans, to be less active, to show less exploratory behaviors, and to be more social compared to control horses. However, contrary to our expectations, we did not find any differences in these traits between stereotypic and control horses. Since reactive coping strategies are characterized by freeze responses and unresponsiveness, it might be more difficult to detect fear in these animals (81). Surprisingly, however, we found that a greater proportion of crib-biters reacted to the tactile filaments compared with control horses, suggesting a higher tactile sensitivity in crib-biters, which to our knowledge has never been reported before. We suggest that this higher tactile sensitivity could be due to altered dopamine or endogenous opioid physiology, resulting from chronic stress exposition. We conclude that it might be valuable to conduct further investigations to assess the personality of stereotypic horses, as it could help to identify genetic loci associated with stereotypies.

### Reactivity to Humans

We did not find any difference between crib-biters and controls in their propensity to react to a passive human. Because this trait has been previously related to boldness, a characteristic of proactive individuals (64, 65), we expected crib-biters, as potential reactive individuals, to interact less with unfamiliar humans. Since how animals react to humans is known to be heavily influenced by the environment (e.g., previous human handling) (82), genetic predisposition acting on this trait might be difficult to detect. We thus suggest that environmental components could have influenced previously existing differences between stereotyped and control horses in our study.

### Tactile Sensitivity

In our *tactile sensitivity test*, a greater percentage of crib-biters reacted to the tactile filaments compared with the control horses. This suggests that crib-biters might be highly sensitive to tactile stimulation. Tactile stimuli stimulate skin receptors also called mechanoreceptors. This information is then transmitted via the spinal cord to the thalamus and on to cortical sensory areas. Tactile information is mapped onto the primary and secondary *somatosensory cortex*. This cortex shows a somatotopic organization, with the most sensitive parts of the body occupying the most cortical territory (83). Difference in sensitivity to tactile stimuli has been reported in some human developmental disorders, such as autism (83). For instance, autistic people with Asperger syndrome are often described as being easily disturbed by their environment because they perceive external stimuli with higher intensities than other people. Some studies also report hypersensitivity to senses, such as touch, smell, and taste in these people (84). Existing theories suggest that this hypersensitivity is due to enhanced processing of stimuli details in the secondary *somatosensory cortex*, or impairment of top-down modulation of incoming stimuli (85, 86). In other mood disorders such as “depression.” on the other hand, “unresponsiveness” to environmental stimuli (tactile or visual) have been reported in both human (67) and animals [monkeys (87), horses (68)]. Tactile sensitivity might therefore be an important indicator of developmental or mood disorders.

A distinction can be made between sensory processing and sensory sensitivity, since individuals can perceive stimuli and not respond to them. Therefore, the hypersensitivity that we observed in crib-biters could be explained firstly by the fact that some horses might feel a tactile stimulation without responding to it. We could hence suggest that control horses might have felt the stimulation, while being less disturbed by it than crib-biters. On the other hand, crib-biters, because of their higher stress sensitivity reported in our previous study (28), might be easily irritated by tactile stimuli and may took a longer time to habituate to them than the controls, as suggested for hypersensitive people (88). It would be interesting to conduct further experiments testing the sensitivity of crib-biters within other senses [e.g., gustato-olfactory, auditory and visual sensitivity; (62)].

The hypersensitivity found in crib-biters could otherwise be explained by neural differences between these horses and controls. We could suggest that exposition to chronic stress may cause alteration of dopaminergic systems, not only in the *mesoaccumbens* dopamine system as reported in stereotypic animals (2, 89), but also in dopaminergic nerve cells implicated in sensory sensitivity (90). Therefore, the dopaminergic modulation impairment that crib-biters potentially suffer from could also be implicated in their sensory hypersensitivity (91). We could hence suggest that the hypersensitivity that we found in crib-biters is explained by differences between stereotypic and non-stereotypic horses in their neural processing of tactile stimuli or in dopamine modulation (5, 57).

An object pressed against the skin can produce various kinds of perception, such as “pain,” “tickle,” or “touch” (72). Similarly, the application of different forces on the skin using von Frey filaments could produce different sensations. Although the exact sensation produced by these filaments remains unknown (71), von Frey filaments are considered as a good method for assessing nociceptive thresholds [in rats (92), in horses (93, 94)]. Differences in β-endorphin physiology has been reported to be implicated in the causal and/ or functional aspect of stereotypic behaviors. Indeed, administration of μ opioid receptor antagonists to different species (dogs, pigs, cats, chickens, horses, and bank voles) has been shown to reduce the performance of stereotypies (4, 95, 96), Even if measurements of plasma β endorphin in crib-biting horses has produced conflicting results (69, 97, 98), a recent study aimed at reassessing opioid physiology in these horses found an upregulation of μ opioid receptors in some part of the *mesoaccumbens* pathway (99). Because endorphins play a role in assessing pain and analgesia, we could hence suppose that the hypersensitivity we found in crib-biters is related to differences in endorphin modulation between the two groups. In alignment with this hypothesis, crib-biting behavior has previously been associated with a decrease in nociceptive (thermal) threshold during crib-biting periods (69). It would be interesting in future studies to investigate opioid receptor sensitivity in stereotypic and normal horses. To summarize, we could thus suggest that the hypersensitivity that we found in crib-biters is related to differences between stereotypic and non-stereotypic horses in their neural processing of pain.

### Social Reactivity

We did not find any difference between crib-biters and controls in their social reactivity, suggesting that crib-biters are not more aroused than control horses when hearing unknown horses, according to our hypothesis. If crib-biters indeed display a reactive coping strategy, these results might contradict findings in pigs, which showed that reactive pigs could be more social (66).

### Locomotor Activity

The data used to score the “locomotor activity” in the *passive human test*, the *social motivation test*, and the *novel object test* did not reveal any difference between crib-biters and controls in this trait. According to previous studies, we expected crib-biters, if they indeed behave as reactive individuals, to show less locomotor behavior than control horses (26, 64). On the other hand, in stereotypic animals of other species [e.g., mice (25, 100) and rhesus macaques (22)], higher incidence of stereotypy development have been associated with more activity. Yet, discrepancies between these studies and ours could be explained by differences in the type of stereotypy displayed (locomotor stereotypies vs. oral stereotypy in our case), by species or experimental protocol differences. Indeed, the measured phenotype of individuals will depend on the initial definition and use of each trait and on the terminology used to define personality, which varies widely between studies (14, 64).

### Curiosity/Fearfulness

According to our previous results showing that crib-biters display physiological characteristics of reactive individuals, i.e., high HPA-axis reactivity (28) and to the results of Nagy et al. (56), we expected stereotypic horses to also display behavioral characteristics of reactive individuals, such as being less fearful (or anxious) than control horses. However, we did not find any difference between our two groups in their fear reaction to the sudden opening of the umbrella. Previous studies showed that crib-biters seem to be less reactive while restrained with a lip-twitch, but to react more strongly to a rapidly inflating balloon compared to non-stereotypic horses (101). Our results did not confirm these results. Discrepancies between these studies and ours could be explained, once more, by experimental protocol differences (102).

We could also suggest that there might exist a difference in fearfulness between crib-biters and non-stereotypic horses, but that the behavioral indicators that are generally used to assess fearfulness are not appropriate to detect such differences between reactive and proactive individuals. Indeed, behavioral reactions to fear-induced reactions might be less strongly expressed (e.g., characterized by freeze responses and unresponsiveness) in those individuals compared to proactive ones (26, 81). Therefore, if crib-biters are really more reactive than other horses, it is possible that, despite a stronger physiological reaction to the opening of the umbrella, their behavior did not change (81). It would thus be useful, in further studies investigating differences in fearfulness between proactive and reactive animals, to measure other types of fear indicators [e.g., physiological responses, Equine Facial Action Coding Systems (FACS)] (103) in addition, in order to increase the accuracy of fear assessments.

In the same way as for the reaction to the opening of the umbrella, we did not find any difference in reaction toward the novel object between crib-biters and controls. Links between reactions to novel objects and stereotypies have also been tested in species other than horses. Unlike in our study, stereotypic mice show greater reactivity, quicker time to approach novel objects and increased object manipulation, suggesting less fearfulness or higher levels of curiosity compared to control mice (25). Similarly, rhesus macaques that express a higher rate of motor stereotypic behavior in captivity are characterized by more frequent contacts with a novel object, indicating higher levels of curiosity than other monkeys (22). Differences between these studies and ours might be due to the type of stereotypies investigated in mice and rhesus macaques, which was, unlike in ours, locomotor (22, 25). We suggest that some similarities with these studies could be found in weaving more than crib-biting horses.

## Conclusion

Our results suggest that crib-biters are more sensitive to tactile stimulation than non-stereotypic horses. This suggest that this higher tactile sensitivity could be also one of the underlying causes of their higher stress sensitivity (28), which might result in the development of stereotypic behavior in these individuals. We also suggested that this higher tactile sensitivity could be due to altered dopamine or endogenous opioids physiology, resulting from chronic stress exposition. On the other hand, we did not find any personality traits that are characteristic of reactive coping individuals in crib-biters, as we had expected (i.e., to be less fearful, to interact less with unfamiliar human, to be less active, to show less exploratory behaviors and to be more social). We suggest that further studies investigating differences in fearfulness between proactive and reactive animals, which in our case were expected to reflect differences between control and stereotyped horses, should include further behavioral and particularly physiological measures. Indeed, this might to help detect differences between proactive and reactive coping strategies, since fear-induced behavioral reactions might be less strongly expressed in reactive individuals compared to proactive ones (26), as recently suggested by Squibb et al. (81). We conclude that further investigations are required to fully characterize the personality of stereotypic horses. This could allow an early detection of individuals prone to develop stereotypies, and hence might help to prevent them to develop this abnormal behavior.

## Ethics Statement

This study was carried out in accordance with the recommendations of the Swiss Federal Veterinary Office. The protocol was approved by the Swiss Federal Veterinary Office (approval number VD 2677 bis; Switzerland).

## Author Contributions

SBF and SB carried out the experiment. DB scored the videos. SBF scored the videos, wrote the manuscript and performed the statistical analyses. EB, KZ, and IB participated to design an edited the manuscript. EB supervised the project.

### Conflict of Interest Statement

The authors declare that the research was conducted in the absence of any commercial or financial relationships that could be construed as a potential conflict of interest.
